# Efficacité de la gestion de vaccins et qualité de vaccination à l'antenne PEV Kisangani en République Démocratique du Congo

**DOI:** 10.11604/pamj.2017.27.4.11513

**Published:** 2017-05-02

**Authors:** Matthieu Betofe Labama, Eugène Basandja Longembe, Joris Losimba Likwela

**Affiliations:** 1 Service Logistique de Vaccination à Kisangani, Democratic Republic of Congo; 2 Faculté de Médecine de l’'UNIKIS; 3 Département de Santé Publique à la Faculté de Médecine et de Pharmacie de l’'UNIKIS

**Keywords:** Gestion des vaccins, chaine d´approvisionnement, vaccins, Vaccine management, supply chain, vaccines

## Abstract

**Introduction:**

La qualité des vaccins conditionne les résultats attendus de la vaccination. Elle est tributaire de l'efficacité de système de gestion technique et logistique mis en place. Cette étude est menée pour évaluer l'efficacité de la gestion des vaccins et d'en tirer des leçons.

**Méthodes:**

Une étude rétrospective est menée pendant la période de 2010 à 2014 sur la gestion logistique de vaccins au niveau de l'antenne PEV Kisangani. La revue documentaire complétée par les entretiens semi-dirigés des gestionnaires et prestataires de services de vaccination ont permis d'évaluer la gestion de vaccins en se servant des modèle GEV de l'OMS, en vue de dégager les écarts.

**Résultats:**

Il est observé une faible connaissance des prestataires sur les vaccins qui ne peuvent pas être congelés, sur les tests de congélation et d'autres dommages de vaccins. La gestion informatisée des données au niveau de l'antenne est correctement assurée. Aucun critère évalué n'a atteint l'objectif de 80%. Le respect de la température de stockage est de 70% au niveau de l'antenne ; le critère relatif à la gestion de vaccins est respectivement de 65% et 67% au niveau du BCZ et CS. Le critère relatif à la maintenance est nul à tous les niveaux.

**Conclusion:**

Le dysfonctionnement de système logistique est remarquable à tous les niveaux de la pyramide sanitaire, ceci pourrait interférer avec la qualité et l'impact attendu de la vaccination. Une attention particulière doit être accordée à la maintenance des équipements.

## Abstract (en)

**Introduction:**

The quality of vaccines is critical for good immunization outcomes. It is dependent on the effectiveness of technical and logistics management system put in place. This study aimed to assess the effectiveness of vaccine management and to learn something from this.

**Methods:**

We conducted a retrospective study of logistic management of vaccines in the Expanded Programme on Immunization (EPI) antenna in the Kisangani region over the period 2010-2014. A literature review completed by semi-structured interviews of immunization services managers and providers helped to evaluate vaccine management using the WHO GEV model in order to identify the gaps.

**Results:**

Provider's demonstrated low level of knowledge of the vaccines that cannot be frozen, of freezing tests and of other vaccine damages. Computerized data management was properly ensured in the antenna. No evaluation criteria reached the goal of 80%. Compliance with the storage temperature was 70% in the antenna; the criterion for vaccine management was 65% and 67% in the health area central office and in the health centre respectively. The maintenance criterion was zero at all levels.

**Conclusion:**

Malfunctioning of the logistic system is remarkable at all levels of the health pyramid; this could interfere with the quality and the expected impact of vaccination. Particular attention should be paid to the maintenance of the equipment.

## Introduction

La vaccination constitue une composante essentielle du droit humain à la santé (individuelle, collective et gouvernementale) et doit être reconnue comme telle. Elle est une des stratégies les plus efficaces pour la réduction de la morbi-mortalité liées aux maladies infectieuses, elle constitue une des interventions de santé publique les plus rentables et représente un investissement essentiel pour l'avenir d'un pays. La vaccination prévient environs 2,5 millions de décès chaque année dans le monde [1]. L'analyse de la situation liée à la gestion de la chaine d'approvisionnement des produits immunisant présente des nombreuses insuffisances qui nécessitent de l'amélioration à tous les niveaux de la pyramide sanitaire [2]. Une attention particulière doit être accordée à sa gestion [3], car elle est plus complexe et préoccupante pour la santé publique mondiale et particulièrement pour les pays de l'Afrique Sub-saharienne [4]. Etant qu'un produit biologique, le vaccin est un intrant périssable, sensible et perd son efficacité s'il n'est pas correctement conservé. Sa bonne gestion est un gage de la performance du programme et fait partie des aspects les plus importants dans la lutte contre la maladie à tous les niveaux de la pyramide sanitaire [5]. Un approvisionnement en produit de qualité, dans une chaine de froid garantie, pérennise le service de vaccination. Pour ce faire, l'optimisation de la logistique est une stratégie efficace pour anticipation aux épidémies (situations d'urgence) et au renforcement de la surveillance, basée sur des données solides [6]. En République Démocratique du Congo (RDC), le système gestionnaire des vaccins s'échelonne sur trois niveaux de la pyramide sanitaire (centrale, intermédiaire et périphérique). Les principaux problèmes opérationnels constatés dans le domaine de gestion de la logistique de vaccination sont: les insuffisances d'utilisation des nouvelles technologies dans l'estimation des besoins, les faiblesses dans la chaine de livraison et de stockage des vaccins, dans le monitorage de la chaine de froid, dans la gestion de la maintenance du parc et dans l'utilisation des logiciels de gestion des données des vaccins et de vaccination. Ces problèmes constituent la cause principale des pertes élevés de vaccins et des ruptures fréquentes de stocks, avec un impact négatif sur la performance des services de vaccination au niveau opérationnel [7]. Ces mêmes problèmes se répercuteraient à l'antenne PEV Kisangani, dont l'ampleur et la répartition de responsabilité suivant les différents critères de GEV ne sont pas connues et non plus documentées. Pour cerner ces problèmes opérationnels, cette étude est menée pour évaluer l'efficacité de la gestion des vaccins au niveau de l'antenne PEV de Kisangani conformément aux normes établies par l'OMS et en tirer des leçons.

## Méthodes

Il s'agit d'une étude descriptive rétrospective sur la qualité de la chaine logistique de vaccins, de l'entrepôt de l'antenne jusqu'au niveau opérationnelle (BCZS et FOSA). La population de notre étude est constituée de toutes les unités de vaccination et de gestion logistique du niveau intermédiaire et périphérique. La collecte de données a été réalisée par convenance au niveau de l'antenne PEV de Kisangani et dans 10 Zones de Santé tirées de manière aléatoire simple sur la liste exhaustive des ZS desservies par l'antenne PEV de Kisangani, au nombre de 17. Au niveau de chaque ZS tirée, le site de stockage du BCZ était d'office retenu et 2 Centres de Santé avec matériels de chaine de froid étaient tirés de manière aléatoire simple. Ceci a ramené la taille de notre échantillon à 31 structures. L'outil/grille d'évaluation GEV développé par l'OMS pour les évaluations quantitative et qualitative des opérations logistiques, a été utilisé pour visualiser les couvertures réalisées par intervention, identifier les goulots et proposer des actions appropriées. Les neuf critères utilisés sont en rapport avec : les approvisionnements, la gestion des stocks, l'entreposage, la manipulation, l'entretien courant de l'équipement, le transport, la surveillance des températures et la gestion des informations sanitaire.

Voici l'illustration: E2: tous les vaccins et diluants sont stockés dans les plages de températures recommandées par l'OMS. E3: les capacités de stockage au froid, à température ambiante et de transport sont suffisantes pour tous les vaccins et consommables requis pour le programme y compris pour les nouveaux vaccins. E4: Les bâtiments, équipements et systèmes de transport permettent un bon fonctionnement de la chaîne d'approvisionnement. E5: la maintenance des bâtiments, des équipements de la chaîne du froid et des véhicules est satisfaisante. E6: la gestion des stocks ainsi que les procédures y afférant sont efficaces. E7: la distribution entre les niveaux de la chaîne d'approvisionnement est efficace. E8: les politiques appropriées de gestion des vaccins en vigueur sont appliquées. E9: les systèmes d'information et fonctions d'appui à la gestion sont satisfaisants (critère non-applicable au niveau centre de santé).

**Evaluation des critères:** selon le contexte de notre étude, nous avons modifiés d'autres paramètres de cette grille d'évaluation. Chaque élément/critère renseigne sur l'efficacité du système gestionnaire des vaccins dans la chaine d'approvisionnement : niveau très faible (0-25%), niveau faible (26-50%), niveau moyen (51-75%), niveau élevé (76-100%). 8 critères d'évaluation (E2 à E9) sont utilisés et scorés. Nous avions procédé par la revue documentaire des différents supports de gestion complétée par l'interview de personnes impliquées dans la gestion de la chaine logistique et l'observation directe de mode de gestion des intrants et matériels de chaine de froid. Les données collectées ont été encodées et traitées à l'aide de logiciel EVM_Assistant_Tool institué par l'OMS, complété par Epiinfo3.5.4 pour les analyses et croisement des variables. Les résultats sont présentés sous forme des graphiques en surface ou radar et tableaux. Les statistiques portent essentiellement sur les proportions et moyennes avec calcul de la déviation standard.

## Résultats

**Critère E2: suivi de température de conservation.** De 24 indicateurs évalués, 12 ont atteint la satisfaction soit 50%, avec la déviation standard de 1,4. 71% d'équipements disposent de fiches de température pour 5 années d'évaluation dont aucune des fiches de suivi de température n'a notifié des alarmes. Des très faibles performances sont notées sur la connaissance par des prestataires des vaccins qui peuvent être endommagés à une température hors plages de < 0°C (29,2%). De la lecture du fridge-tag (moniteur automatique de suivi de température) au jour de l'enquête, il est noté la concordance entre les enregistrements des graphes et les relevés manuels à 3,2% et pour la révision des enregistrements de température pour identification des excursions et de leurs causes à 0%. Notons qu'aucune structure ne dispose des dispositifs de suivi de la chaine de froid par téléphonie cellulaire (SMS alarme/testo).

**Critère E3: évaluation de la capacité de stockage en froid, de magasin et de transport.** De 24 indicateurs du critère évalués, 6 ont atteint la satisfaction souhaitée soit 25%, avec une déviation standard de 1,4. Il est observé que 100% des entrepôts disposent des conteneurs passifs et 81,8% disposent d'un espace de stockage des consommables. La couverture en matériel solaire est de 13%.

**Critère E4: fonctionnement de la chaine d'approvisionnement.** La performance observée est de 53% soit 32 sur 60 indicateurs évalués, avec une déviation standard de 1,3. Les éléments qui font défaut sont entre autres: la disponibilité d'extincteurs d'incendie, le local ventilé et le magasin sec avec étagères/palettes bien organisées et positionnées ainsi que de dispositif pour le prélèvement de l'humidité. Les enregistreurs continus de températures dans les équipements n'existent qu'à 3,2% et la disponibilité des sources d'énergie (kérosène et générateur) n'est qu'à 29%.

**Critère E5: système de maintenance préventive du parc.** Tous les indicateurs de maintenance sont nuls.

**Critère E6: procédures de gestion des stocks.** Il est noté que la performance observée pour ce critère est très faible (17%) soit 15 sur 87 indicateurs évalués avec une déviation standard de 1,2. La gestion physique des stocks demeure un réel problème dans le système logistique du PEV Kisangani. Tous les indicateurs d'enregistrement, de rapportage et des transactions sont quasi nuls. La gestion informatisée des données (utilisation des logiciels SMT et DVD-MT) s'effectue qu'au niveau de l'entrepôt de l'antenne. Le suivi des stocks critiques ne se réalise presque pas, la gestion s'effectue au quotidien et entraine des ruptures fréquentes dans 64,5% des structures. Les établissements avec mesure d'approvisionnement des sites de vaccination, les entrepôts avec stock en vaccins sécurisé, correctement stockés et la sortie selon l'ordre PEPS, existent à 100%.

**Critère E7: système de distribution entre les niveaux de la chaine d'approvisionnement.** Il sied de noter que la performance observée pour ce critère est de 29% soit 13 sur 45 indicateurs évalués avec une déviation standard de 1,3. Les bonnes performances sont observées en rapport avec le nombre de livraisons planifiées et exécutées, la préparation et le conditionnement d'accumulateurs de froid. On note que l'antenne ne dispose d'un entrepôt mobile, ni d'une véhicule frigorifique.

**Critère E8: évaluation des politiques appropriées de gestion des vaccins.** De 51 indicateurs évalués, 30 ont réalisés un score acceptable soit 59% avec une déviation standard de 1,5. Les indicateurs lacunaires observés sont notamment sur la connaissance de différents tests de congélation et d'autres dommages, les instructions écrites sur l'interprétation de PCV/VVM, la gestion des flacons entamés (politique des flacons entamés), les données complètes sur les pertes et l'exploitation des données pour le suivi de performance.

**Figure 1:**
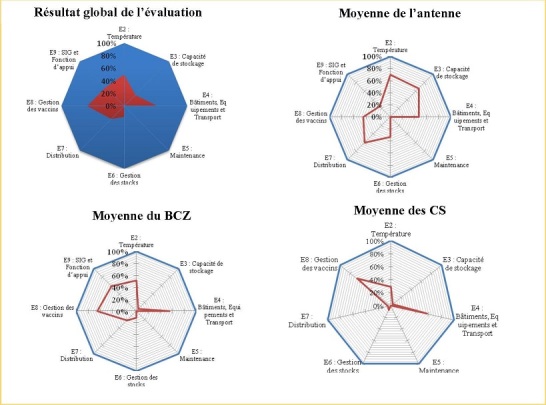
Présentation de résultat de l’évaluation par niveau de la pyramide sanitaire

**Critère E9: évaluation du système d'information logistique et les fonctions de soutien.** La performance observée est de 42% soit 15 sur 36 indicateurs évalués avec une déviation standard de 1,5. Les valeurs sont quasi nulles dans les composantes : mises en place des procédures opératoires normalisées et de plan annuel de travail. Quelques bonnes performances sont observées dans la collecte et l'utilisation des données du terrain pour la prise des actions (Tableau 1 , Figure 1).

**Tableau 1:** Résultat global par critère et par niveau de la chaine d’approvisionnement

Critères de la GEV	Objectif	Scores moyen	Niveau de la chaine		
			Antenne	ZS	CS
E2: suivi de températures de stockage correctes des vaccins	**80%**	**50%**	70%	51,2%	28,6%
E3: maintien de capacités de stockage et de transport suffisant	**80%**	**25%**	66,6%	5%	3%
E4: bâtiments, Equipements et Transport	**80%**	**53%**	47%	56%	57%
E5: maintenance	**80%**	**0%**	0%	0%	0%
E6: gestion des stocks	**80%**	**17%**	32,6%	11%	8%
E7: distribution	**80%**	**29%**	60%	22%	5%
E8: gestion des vaccins	**80%**	**59%**	44,4%	65,2%	67%
E9: SIG et Fonction d’appui	**80%**	**42%**	25%	58,7%	-

## Discussion

Les résultats d'évaluation des critères E2 à E9 observés dans cette étude sont inférieurs à la moyenne acceptable de 80%. Au niveau intermédiaire (antenne), le score le plus élevé est de 70% pour le critère relatif à la température de stockage (E2); au niveau périphérique (Zone de Santé) le score maximum est de 65%, relatif à la gestion des vaccins (E8); au niveau de prestation de service (centres de santé), pour les critères E2-E8 évalués, le score maximum obtenu au critère E8relatif à la gestion des vaccins est de 67%. A tous les niveaux, le critère relatif à la maintenance est nul (0%). Parmi les principales faiblesses observées dans cette étude, il y a l'insuffisance de monitorage de température, l'inexistence du calendrier des arrivages et d'un logiciel programmatique des approvisionnements à l'antenne, la gestion de vaccins et de chaine de froid non informatisée, la faible capacité de stockage, l'insuffisance de la sécurisation physique des entrepôts, la faible couverture en chaine de froid solaire, la faible disponibilité en sources d'énergie, l'inexistence de plan de maintenance préventive des infrastructures et des équipements de la chaîne du froid, la faible capacité de gestionnaires des dépôts dans la réalisation des tests de congélation et d'autres dommages de vaccins, l'absence des procédures opératoires normalisées à tous les niveaux. Des bonnes performances sont notées en rapport avec la gestion informatisée des données au niveau de l'antenne et les efforts croissants vers l'atteinte de l'objectif en rapport avec le respect de la température de stockage au niveau de l'entrepôt. Ces résultats sont moins bons que ceux trouvés au cours d'une étude menée en 2011 au TOGO [8]. Au niveau régional, un critère sur les 8 a atteint le score de 80%, il s'agit du système d'information et d'appui à la gestion (E9). Par contre le niveau périphérique (ZS) avait présenté un profil globalement satisfaisant avec 3 critères sur les 8 évalués qui ont un score de plus 80%.Au niveau d'Unité de Soins Périphérique (CS) aucun critère n'est satisfaisant sur les 7 évalués. Le plus bon score à ce niveau est réalisé par le critère E8 relatif à la gestion des vaccins et consommables (87%), tandis que le critère E6 relatif à la gestion des stocks est le plus faible avec (62%).

Par contre, nos résultats corroborent avec ceux trouvés en République de Côte d'Ivoire où, au niveau régional, aucun critère n'a atteint le score de 80% exigé. Seule la catégorie de formation a atteint l'objectif avec 88% [9]. Des faiblesses sont constatées dans les catégories suivantes: bâtiments vétustes, faible capacité de stockage due essentiellement au manque de magasin sec, documentation défectueuses, faiblesse dans la maintenance préventive, faible couverture en matériels solaire, non fonctionnalité de générateurs électriques. Certaines de ces faiblesses influent négativement sur la qualité de vaccins par le fait des ruptures fréquente des fournitures en énergie. Au niveau de district (ZS) les catégories formation et capacité de stockage ont atteint l'objectif de 80% et au niveau centre de santé, seul le critère « distribution », a atteint l'objectif de 80%. L'évaluation de la gestion de la chaine logistique des vaccins aux iles COMORES nous révèle aussi, au niveau régional [10], des insuffisances avec un seul critère qui présente un score satisfaisant de 97%, le critère E9 relatif aux systèmes d'information et fonctions de soutien. Les autres critères varient d'un score de 51% (E5) à 72% (E8). Au niveau de district (ZS), seul le critère E3relatif aux capacités de stockage présente un score satisfaisant de 91%, les autres critères varient d'un score de 52% (E7) à 78% (E8).Au niveau des prestations de service, les résultats obtenus varient entre88% (E3: capacités de stockage) et 52% (E5: maintenance). Néanmoins, ces résultats semblent assez améliorer que les nôtres bien que des grands soucis persistent. Une évaluation récente réalisée en République Démocratique du Congo par le Ministère de la Santé Publique, dans les 26 antennes de PEV, pour la période de 2011-2014 [11], a révélé qu'un seul critère (E8) relatif aux connaissances des agents et mise en œuvre des politiques de gestion des vaccins était satisfaisant avec un score de (85%) et tous les autres critères étaient en dessous de l'objectif des 80%. Le score en rapport avec la capacité de stockage (infrastructures, équipements), le transport et la distribution à ce niveau est autour de 60%. Ces données sont similaires à celles observées au niveau régional de TOGO avec respectivement les scores de 48% dans le domaine de la distribution des vaccins, 61% des bâtiments et équipements et 63% les capacités de stockage [8]. Il était par ailleurs observé au niveau de 26 Zones de Santé en RDC et de prestation de service que, seul le critère E8 était autour de 80%. Ce qui indique que les acteurs à ces deux niveaux ont des compétences et connaissances en matière de gestion des vaccins, contrastant avec les scores très faible de certains critères tels que la maintenance (21%) et la gestion des stocks (33%). Il va de soi que la maintenance et la gestion de stock constituent les principaux goulots à ces niveaux de la pyramide sanitaire.

Il ressort d'une étude menée au Cameroun que des agents de santé possèdent des bonnes connaissances sur la durée de stockage des vaccins dans les établissements de santé, mais des faiblesses ont été observées en rapport avec le placement des vaccins contre la rougeole au réfrigérateur [12]. Il était ainsi recommandé, pour pallier à ses insuffisances, de renforcer les compétences des agents en termes de formation et supervision périodique [13]. La gestion des stocks de vaccins est confrontée à certains problèmes dont essentiellement le non-respect de la température de stockage, la faible capacité de stockage de la chaine de froid (CdF), l'absence des infrastructures et équipements de la CdF et du transport, l'absence de plan de maintenance de la CdF, la mauvaise tenue de gestion des stocks de vaccins. Ceci rejoint les résultats d'une étude menée au Benin en 2008 qui a montré l'exposition des vaccins à des températures supérieurs à +8°C dans 77% des formations sanitaires et l'exposition à des températures inférieures à moins 5°C dans 55% de formations sanitaires pendant cinq jours [14]. Pour garantir la qualité des vaccins, les thermomètres Testo permettent une surveillance plus efficace de la chaine du froid et une meilleure réactivité pour la correction des anomalies détectées [15]. La surveillance informatisée de la température de la chaîne du froid offre un meilleur moyen de suivi des indicateurs de congélation, la Pastille de Contrôle de Vaccins et les bandes colorées pour suivre les températures de conservation des vaccins PEV, en particulier les nouveaux vaccins, plus sensibles à la chaleur et à la congélation.

En Afrique sub-saharien, les difficultés de gestion de stocks ont été également notées par différents auteurs au cours d'études d'évaluation de gestion de vaccins. On note le renouvellement du stock après épuisement du stock dans 14,3% des cas, le mode de gestion physique des stocks a été la gestion au quotidien, conséquence d'une rupture de stock dans 62,9% des formations sanitaires [16]. Ceci corrobore avec les résultats de notre étude. Les contre-performances observées sur plusieurs critères dans cette étude est une réalité de tous les pays en voies de développement. Ceci se justifierait par la similarité de cadre organisationnel et fonctionnel de leurs systèmes de santé confrontés aux problèmes d'insuffisance de ressources pour l'offre de cadre adapté, la mise à disposition des équipements appropriés et leurs maintenances, l'insuffisance de renforcement de capacité des prestataires et leurs faible rémunération, l'accès limité à l'information, les barrières géographiques, culturelles et financières qui réduisent l'accès aux services de santé de qualité. En réponse aux contraintes de stockage et aux différents goulots d'étranglement lié à la chaîne d'approvisionnement, l'augmentation de la capacité de stockage par des nouvelles dotations en équipements de chaine de froid solaire peut, dans certains cas, répondre à la demande croissante en raison de la croissance démographique, des nouvelles introductions de vaccins et des emballages plus importants [17]. La modélisation dynamique peut fournir un aperçu de l'endroit où ces ressources peuvent sauver la plupart des vies [12, 18].

## Conclusion

Les évaluations quantitative et qualitative des opérations logistiques basées sur les critères définis par l'OMS dans les pays en voie de développement montrent de manière générale, des lacunes sur l'ensemble des critères, malgré les succès sporadiques observés de manière disparate pour certains critères et entre les pays. En dépit des efforts orientés vers l'augmentation de la couverture vaccinale, une attention particulière doit être attirée vers l'amélioration des opérations logistiques, garant de la qualité de vaccins et de service de vaccination, comme un complément obligatoire en vue de contribuer à la réduction de la morbidité et mortalité dues aux maladies évitables par la vaccination. Le management de la logistique doit désormais, faire partie intégrante de paquet minimum d'interventions en PEV en vue d'accroitre l'efficacité de la chaîne d'approvisionnement des vaccins conformément à la nouvelle technologie de gestion des services de vaccination et aux standards internationaux.

### Etat des connaissances actuelle sur le sujet

- La gestion de vaccins est dit « efficace » si l'ensemble des procédures et des bonnes pratiques d'estimation, d'approvisionnement, de stockage et de distribution permettent de mettre à la disposition des structures sanitaires les vaccins de qualité;
- La qualité des vaccins est garantie par le maintien de la température optimale de +2°C à +8°C pendant l'entreposage et le transport jusqu'au client et l'utilisation de la nouvelle technologie innovante à la matière;
- Pour ce faire, les gestionnaires des structures de santé sont directement responsables du bon fonctionnement de la chaîne du froid et de la bonne gestion de maillons de la chaine d'approvisionnement des vaccins, ainsi que de la visibilité du niveau des stocks pour faciliter la prévision et la planification de la distribution d'une part et de remédier aux excès ou aux ruptures de stock d'autre part.

### Contribution de notre étude à la connaissance

- L'alerte vers l'amélioration des opérations logistiques pour garantir la qualité des vaccins et de service de vaccination, est un complément obligatoire à la contribution de la réduction de la morbidité et mortalité dues aux maladies évitables par la vaccination;
- La proposition de management de la logistique comme partie intégrante de paquet minimum d'interventions en PEV pour accroitre l'efficacité de la chaîne d'approvisionnement des vaccins conformément à la nouvelle technologie de gestion des services de vaccination et aux standards internationaux;
- La maintenance des équipements est le maillon le plus faible de la logistique dans les pays en développement, ce qui ne garantit pas la demi-vie des équipements.

## Conflits d’intérêts

Les auteurs ne déclarent aucun conflits d'intérêts.

## References

[1] Organisation Mondiale de la Santé (2013). Plan d'Action Mondial pour les Vaccins 2011-2020.

[2] Organisation Mondiale de la Santé (2006). GIVS; la vaccination dans le monde: vision et stratégie 2006 - 2015.

[3] OMS, UNICEF, Banque Mondiale (2010). Vaccins et vaccination: la situation dans le monde.

[4] Organisation Mondiale de la Santé (2012). Assurance Qualité des Vaccins de la Réception à l'Utilisateur.

[5] Ministre de la Santé du Canada (2007). Lignes directrices nationales sur l'entreposage et la manipulation des vaccins.

[6] USAID, PROJECT DELIVER (2011). Manuel de logistique : un guide pratique pour la gestion de la chaine d'approvisionnement des produits de santé.

[7] Ministère de la Santé, RDC (2013). Rapport annuel du Programme Elargi de Vaccination (PEV). 2012.

[8] Azondo M (1991). Evaluation de l'impact du processus gestionnaire des stocks de vaccins sur les résultats de l'action vaccination au TOGO. Mémoires de fin d'étude, diplôme supérieur de gestion des services de santé.

[9] Ministère de la Santé et de lutte contre le SIDA, République de Cote d'Ivoire (2012). Evaluation de la Gestion Efficace des Vaccins (GEV) en CI. Rapport d'évaluation.

[10] UNION DES COMORES (2012). Evaluation de la Gestion Efficace des Vaccins (GEV) aux Comores. Rapport de l'évaluation.

[11] Ministère de la Santé Publique, RDC (2014). Évaluation de la Gestion Efficace des Vaccins en République Démocratique du Congo. Rapport de l'évaluation.

[12] Yakum MN, Ateudjieu J, Walter EA, Watcho P (2015). Vaccine storage and cold chain monitoring in the North West region of Cameroun: a cross sectional study. BioMed Central (BMC) Research Notes..

[13] Ateudjieu J, Kenfack B, Wakam NB, Demanou M (2013). Program on immunization and cold chain monitoring: the status in eight health districts in Cameroon. BioMed Central (BMC) Research Notes..

[14] FAYE Moustapha (2008). Evaluations de la gestion des vaccins et consommables vaccinaux du PEV en Afrique de l'Ouest. Mémoire de fin d'études, Diplôme Inter Universitaire (DIU) : Organisation et management des systèmes publics de prévention vaccinale dans les pays en développement »..

[15] Schlumberger M, Mireux F, Tchang SG, Mboutbogol D, Cheikh DO (2011). Surveillance informatisée de la chaîne du froid en milieu tropical (Tchad). Med Trop..

[16] Ministère de la Santé, République du TOGO (2011). Direction PEV TOGO: Évaluation de la Gestion Efficace des Vaccins (GEV) au Togo. Résultats et recommandations.

[17] Leila A, Haidari, Diana L, Connor, Angela R, Wateska (2013). Augmenting Transport versus Increasing Cold Storage to Improve Vaccine Supply Chains. PLOS ONE..

[18] Silve B, Ouedraogo A (2013). Professionalizing Health Logistics in Burkina Faso: Challenges, Implementation and Sustainability. Public Health Research..

